# Prospective, Randomized Comparison of Same-Day Dose of 2 Different Bowel Cleanser for Afternoon Colonoscopy: Picosulfate, Magnesium Oxide, and Citric Acid Versus Polyethylene Glycol

**DOI:** 10.1097/MD.0000000000000628

**Published:** 2015-04-03

**Authors:** Tae-Geun Gweon, Sang Woo Kim, Yong-Sun Noh, Seawon Hwang, Na-Young Kim, Yoonbum Lee, Soon-Wook Lee, Sung Won Lee, Jong Yul Lee, Chul-Hyun Lim, Hyung Hun Kim, Jin Su Kim, Yu Kyung Cho, Jae Myung Park, In Seok Lee, Myung-Gyu Choi

**Affiliations:** From the Division of Gastroenterology, Department of Internal Medicine, Seoul St. Mary's Hospital, The Catholic University of Korea, College of Medicine

## Abstract

For afternoon colonoscopy, same-day administration of sodium picosulfate, magnesium oxide, and citric acid (PM/Ca) is recommended. However, few studies have evaluated the bowel-cleansing efficacy and safety of this regimen. The aim of this study was to compare the bowel-cleansing efficacy, side effects, and patient's tolerability of a same-day split administration of PM/Ca with polyethylene glycol (PEG) for afternoon colonoscopy.

Patients were randomly assigned to a PM/Ca group or a PEG group. The PM/Ca group consumed 1 sachet of PM/Ca at 06:00 and 1 sachet of PM/Ca 4 hours before the colonoscopy. They also took 2 tablets of bisacodyl before sleep on the night before. The PEG group consumed 2 L of PEG at 06:00 and 2 L of PEG 4 hours before the colonoscopy. All subjects were instructed to finish the bowel cleanser or fluid at least 2 hours before colonoscopy. All colonoscopic examinations were performed in the afternoon on the same day. The bowel-cleansing efficacy was scored using 2 scales: the Ottawa Bowel Preparation Scale (OBPS) and the Aronchick scale. Ease of using the bowel cleanser was rated from 1 (very easy) to 5 (very difficult).

Two hundred nine patients underwent colonoscopy. The bowel-cleansing scores by OBPS did not differ between groups (5.0 vs 4.9, *P* = 0.63). Ease of using the bowel cleanser was superior in the PM/Ca group (*P* < 0.01).

The cleansing efficacy of PM/Ca administered on the day of colonoscopy is comparable to that of PEG. Patients prefer PM/Ca.

## INTRODUCTION

Proper bowel cleansing is essential for the precise detection of colon polyps and colorectal cancer.^1,2^ A solution of sodium picosulfate, magnesium oxide, and citric acid (PM/Ca) is a low-volume bowel cleanser comprising a stimulant component and an osmotic component.^3^ Several studies have demonstrated the excellent cleansing efficacy of PM/Ca.^4–7^ PM/Ca also appears to be better tolerated than polyethylene glycol (PEG).^5,6,8^ However, 1 report has shown that electrolyte imbalance must be considered when administering PM/Ca.^9^ PEG is an osmotic laxative used widely as a bowel cleanser. Several studies have shown that PEG is effective in bowel cleansing, and the safety of PEG has been proven in patients with comorbidities such as renal failure, liver disease, and congestive heart failure.^1^ However, ingestion of a large volume of fluid before colonoscopy may reduce tolerability and patient compliance.^10^

Studies have shown that, for afternoon colonoscopy, administration of a bowel cleanser on the same day of the colonoscopy is effective.^11–13^ One study reported that the administration of PEG on the same day of the colonoscopy was more effective than PEG given on the day before.^12^ Another study reported that the bowel-cleansing efficacy was similar for PEG given on the same day of the examination compared with split-dose PEG.^11^ The other study reported superior bowel-cleansing efficacy of PM/Ca administered on the day of the afternoon colonoscopy compared with split-dose PM/Ca.^13^

These previous studies have compared only the same bowel cleanser given in different regimens and did not evaluate safety.^11–13^ No study has evaluated the cleansing efficacy of 2 different bowel cleansers administered on the same day of the afternoon colonoscopy. The European Society of Gastrointestinal Endoscopy recommends a same-day regimen of 4 L of PEG as the standard bowel cleanser and same-day PM/Ca as an alternative regimen for afternoon colonoscopy.^14^ The aim of this study was to compare the bowel-cleansing efficacy, side effects, and patient tolerability of the same-day administration of PM/Ca with that of PEG for afternoon colonoscopy.

## MATERIALS AND METHODS

### Study Population

Enrolment of study subjects was done between May 2013 and September 2013. Individuals who received a colonoscopic examination for various reasons were included in this study. Individuals who met all of the following criteria were included: age 18 to 65 years; outpatient; afternoon colonoscopy; ingestion of the bowel cleanser on the same day of the colonoscopy; and consent to participate in the study. Individuals who met any of the following criteria were excluded at screening: age <18 or >65 years; systolic blood pressure <90 mm Hg or diastolic blood pressure <60 mm Hg; ileus or bowel obstruction; active colitis or inflammatory bowel disease; chronic kidney disease (creatinine clearance <30 mL/min); heart failure (New York Heart Association class III or IV); previous history of bowel surgery; nursing or pregnant; constipation (defecation <3 times per week); unstable angina or myocardial infarction within 6 months; advanced liver cirrhosis (Child–Pugh class B or C); and inpatient.

### Study Design and Procedure

This study was a phase 3, randomized, single-center, assessor-blinded noninferiority study conducted in Seoul St. Mary's Hospital, a university-affiliated tertiary care center in South Korea. After agreeing to participate in the study, the study subjects were randomly assigned to 2 groups: PM/Ca group and PEG group. Randomization was done by a computer-generated randomization list on 1:1 manner. Participants were assigned by sequentially generated randomization list. Coordinator assigned the participants and instructed the bowel-cleansing regimen. Baseline characteristics were checked on the day of the enrolment. Written informed consent was given by all study subjects after randomization.

The study subjects assigned to the PEG group were instructed to take 2 L of PEG at 06:00 on the day of the colonoscopy, and 2 L of PEG was administered 4 hours before the colonoscopy. The study subjects assigned to the PM/Ca group were instructed to take 1 sachet of PM/Ca mixed with 150 mL of water at 06:00 on the day of the colonoscopy. One sachet of PM/Ca mixed with 150 mL of water was administered 4 hours before the colonoscopy. The subjects in the PM/Ca group were encouraged to drink up to 4 L of water or clear fluid (sports drink or honey water). Before sleep on the night before the colonoscopy, the PM/Ca group subjects took 2 tablets of 5 mg bisacodyl. All study subjects were instructed to finish the bowel cleanser or fluid at least 2 hours before the colonoscopy.

Before the colonoscopy, all study subjects completed a questionnaire asking about side effects and tolerability of the regimen. The questions focused on the following: ease of taking the bowel cleanser; willingness to use it again; whether the full dose of the bowel cleanser was ingested; the amount of water and bowel cleanser ingested; any side effects during the ingestion of the bowel cleanser; and the start and finish times of taking the bowel cleanser. The study protocol was approved by the Institutional Review Board of Seoul St. Mary's Hospital.

### Bowel-Cleansing Efficacy

Bowel cleansing was rated by the Aronchick scale and the Ottawa Bowel Preparation Scale (OBPS). The Aronchick scale was graded as follows: excellent (>90% of the mucosa seen, mostly liquid stool, minimal suctioning needed for adequate visualization); good (>90% of the mucosa seen, mostly liquid stool, significant suctioning needed for adequate visualization); fair (>90% of the mucosa seen, mixture of liquid and semisolid stool, could be suctioned and/or washed); poor (<90% of mucosa seen, mixture of semisolid and solid stool, which could not be suctioned or washed); and very poor (repeat preparation needed).^15^

The OBPS was rated by the combination of the total fluid amount and sum of the segmental score.^5^ Total fluid amount was graded on a 3-point scale: 0, minimal; 1, moderate; or 2, large. The colon was divided into 3 segments: right colon, cecum, and ascending colon; midcolon, transverse and descending colon; and (3) left colon, sigmoid colon, and rectum. The score for each colon segment was graded on a 5-point scale as follows: excellent, 0 (perfect preparation; if fluid is present, it is clear and of a minimal amount); good, 1 (mild staining of some turbid fluid or stool but the mucosa is visible without washing and suctioning); fair, 2 (suctioning, but not washing, is needed to see the mucosa); poor, 3 (suctioning and washing are needed); and inadequate, 4 (unable to see the mucosa). The OBPS score ranged from 0 to 14 (fluid score, 0–2; and segment score, 0–12).

Primary endpoint was a total OBPS score. To minimize shortcoming of the OBPS score, success rate assessed by OBPS and Aronchick scale was compared between the 2 groups. Successful cleansing was defined as follows: Aronchick scale: excellent, good, or fair; and OBPS score: fluid score <2 and each segment ≤2. Colonoscopy and scoring of bowel-cleansing efficacy were performed by 3 experienced endoscopists, each with >6 years of experience. The endoscopists were blinded to the bowel cleanser. Before the start of this study, the 3 endoscopists underwent training to use the OBPS and Aronchick scale.

### Secondary Outcomes: Safety, Tolerability, and Polyp and Adenoma Detection Rate

Safety was assessed according to vital signs, laboratory test results, and questionnaire findings. Vital signs were checked on the day of enrolment and after the bowel preparation. Serum hemoglobin concentration, hematocrit, white blood cell (WBC) count, and concentrations of sodium, potassium, chloride, calcium, phosphorus, magnesium, creatinine, and blood urea nitrogen (BUN) were checked after the bowel preparation. The study subjects were asked to choose “yes” or “no” to record whether they had experienced nausea, vomiting, abdominal pain, dizziness, tingling sensation, bloating, or any other symptom during or after the ingestion of the bowel cleanser.

In the questionnaire, the tolerability of the bowel cleanser was assessed according to the ease of use of the bowel cleanser, willingness to use it again, whether the full dose of the bowel cleanser had been ingested, and the amount of ingested water and bowel cleanser. The ease of use of the bowel cleanser was rated by a 5-point scale: excellent, 1; good, 2; fair, 3; poor, 4; and very poor, 5. The polyp detection rate and adenoma detection rate were investigated by colonoscopy and histology.

### Statistical Analysis

The sample size was calculated using the total OBPS score. Assuming the mean OBPS score 5.0 and the standard deviation 2.0 in the PEG group, the significant difference between the 2 groups were hypothesized at 0.6 point. With 80% power and a 2-sided α of 0.05, the sample size in each group was calculated to be 103. Predicting a 10% drop rate, the sample size in each group was 115. All statistical analyses were conducted using SAS 9.0 software (SAS Institute, Cary, NC). The demographic characteristics, bowel-cleansing efficacy, vital signs, laboratory findings, side effects, and tolerability were compared between the 2 groups. For this analysis, we used Student *t* test for continuous variables and the χ^2^ test or Fisher exact test for categorical variables. In the analysis of OBPS score, the overall scores and scores for each segment were compared using Student *t* test. The success rate for bowel cleansing (%) for the 2 groups was calculated as follows: number of individuals with successful cleansing/number of individuals included in the study × 100. The success rate was calculated using the Aronchick scale and OBPS. The success rate was compared between the 2 groups using the χ^2^ test. A *P* value <0.05 was considered significant.

## RESULTS

### Enrolment of the Study Subjects

**FIGURE 1:**
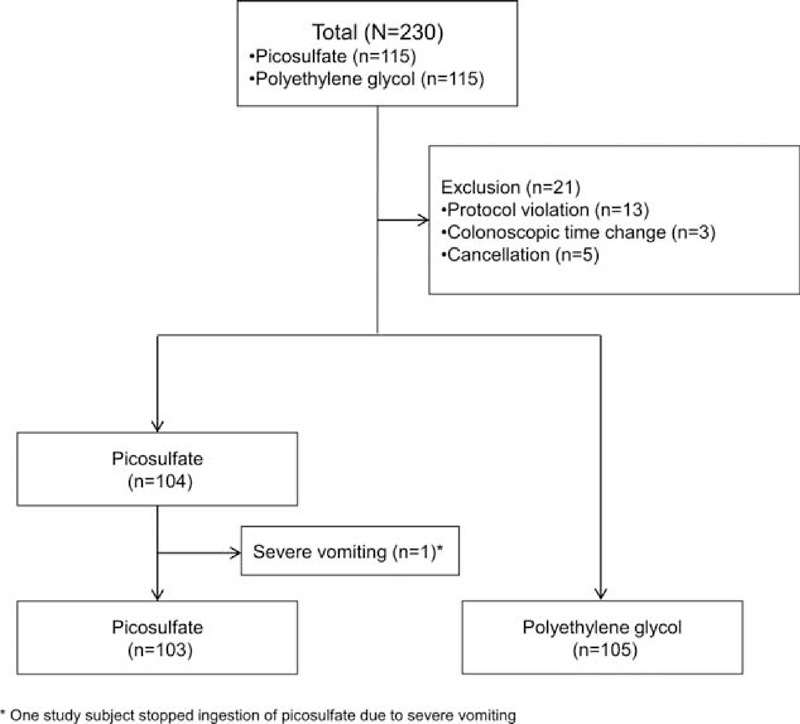
Enrolment of the study subjects.

Among the 230 patients who were enrolled initially, 21 were excluded for the following reasons: individuals who started to ingest the bowel cleanser on the day before the colonoscopy, 13; individuals who changed the colonoscopy time to the morning, 3; and individuals who cancelled colonoscopy, 5. Two hundred nine subjects were included in this study: 105 in the PEG group and 104 in the PM/Ca group (Figure 1). One study subject in the PM/Ca group did not receive the colonoscopy because of vomiting during the ingestion of PM/Ca and 103 subjects from the PM/CA group were included in the analysis of the bowel-cleansing efficacy and laboratory test results.

### Baseline Characteristics of the Study Subjects

The baseline characteristics of the 2 groups are shown in Table 1. Sex, age, and body mass index did not differ between the 2 groups. The indications for colonoscopy differed significantly between the 2 groups (*P* = 0.04).

**TABLE 1:**
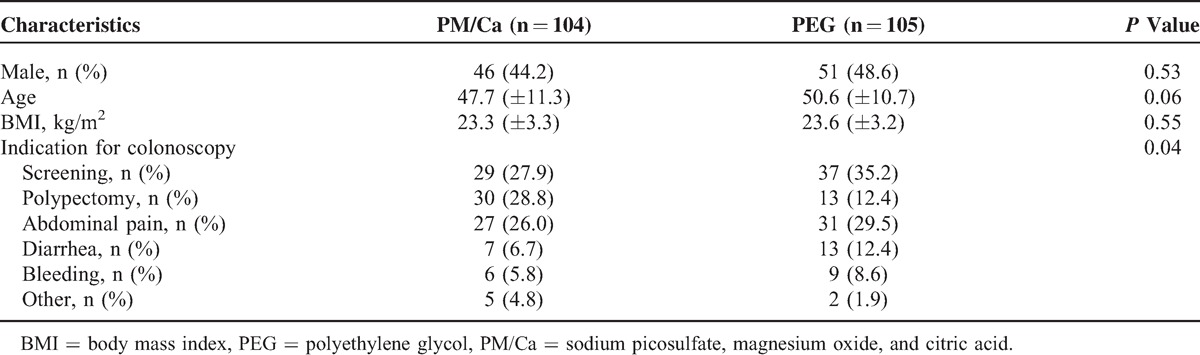
Baseline Characteristics

### Bowel-Cleansing Efficacy

The data for bowel-cleansing efficacy of the 2 groups are shown in Table 2. The total OBPS scores did not differ between the 2 groups (PM/Ca, 5.0 ± 1.7 vs PEG, 4.9 ± 1.7, *P* = 0.63). The total fluid score and regional scores did not differ between the 2 groups. The success rates assessed by the OBPS and Aronchick scale did not differ between the PM/Ca and PEG groups (OBPS, 83.5% vs 84.8%, respectively, *P* = 0.80; Aronchick scale, 95.1% vs 96.2%, respectively, *P* = 0.75).

**TABLE 2:**
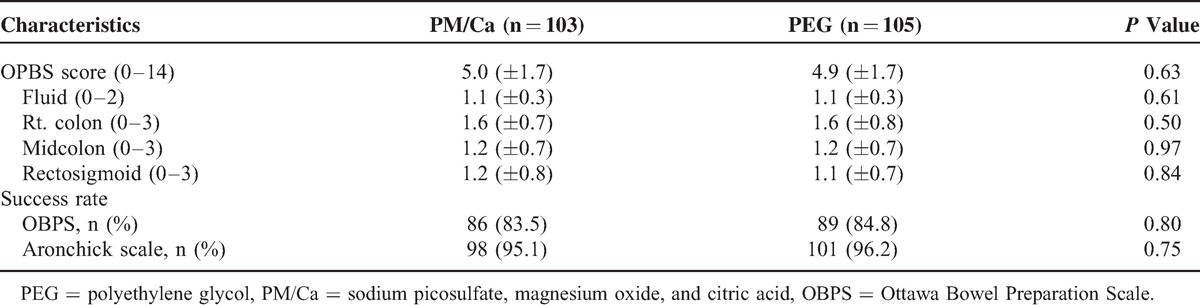
Bowel Cleansing Efficacy in Study Subjects Undergoing Colonoscopy

### Safety

The results of the laboratory tests are shown in Table 3. The hemoglobin concentration, hematocrit, WBC count, and concentrations of creatinine, potassium, calcium, and phosphorus did not differ between the 2 groups. However, the mean BUN was higher in the PEG group than in the PM/Ca group (12.6 ± 3.9 vs 10.4 ± 3.4 mg/dL, *P* < 0.01). Concentration of sodium and chloride was significantly lower in the PM/Ca group than in the PEG group (Na, 137.1 ± 3.9 vs 142.1 ± 2.2 mEq/L, respectively, *P* < 0.01; Cl, 100.0 ± 3.6 vs 103.5 ± 2.7, respectively, *P* < 0.01). Magnesium concentration was higher in the PM/Ca group (*P* < 0.01). All values were within the normal range.

**TABLE 3:**
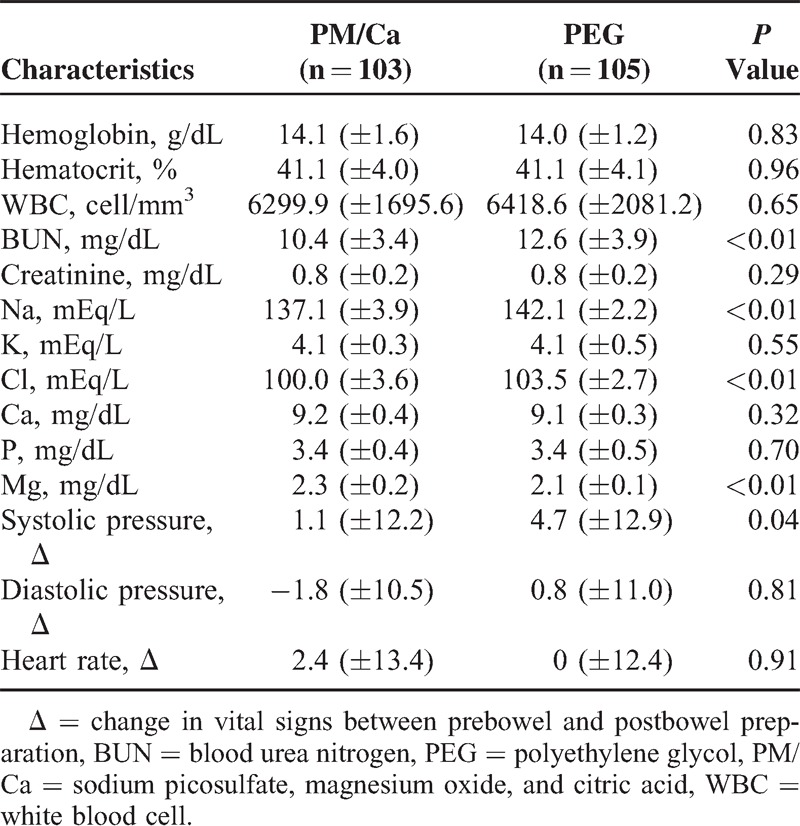
Changes in Vital Signs and Postbowel Preparation Laboratory Tests

After bowel preparation, systolic blood pressure decreased significantly in the PEG group compared with the PM/Ca group (*P* = 0.04). There were no significant changes from before to after the bowel preparation in diastolic pressure and heart rate in either group. Hemodynamic instability (systolic pressure <90 mm Hg or diastolic pressure <60 mm Hg) was not observed in any study subject.

The side effects assessed by questionnaire are shown in Table 4. The number of subjects without adverse symptom was 49 (47.1%) in the PM/Ca group and 45 (42.9%) in the PEG group (*P* = 0.54). More study subjects complained of abdominal pain in the PM/Ca group than in the PEG group (18.3% vs 4.8%, respectively, *P* < 0.01). The number of subjects who reported developing nausea, vomiting, dizziness, tingling sensation, bloating, or other symptoms did not differ between the 2 groups.

**TABLE 4:**
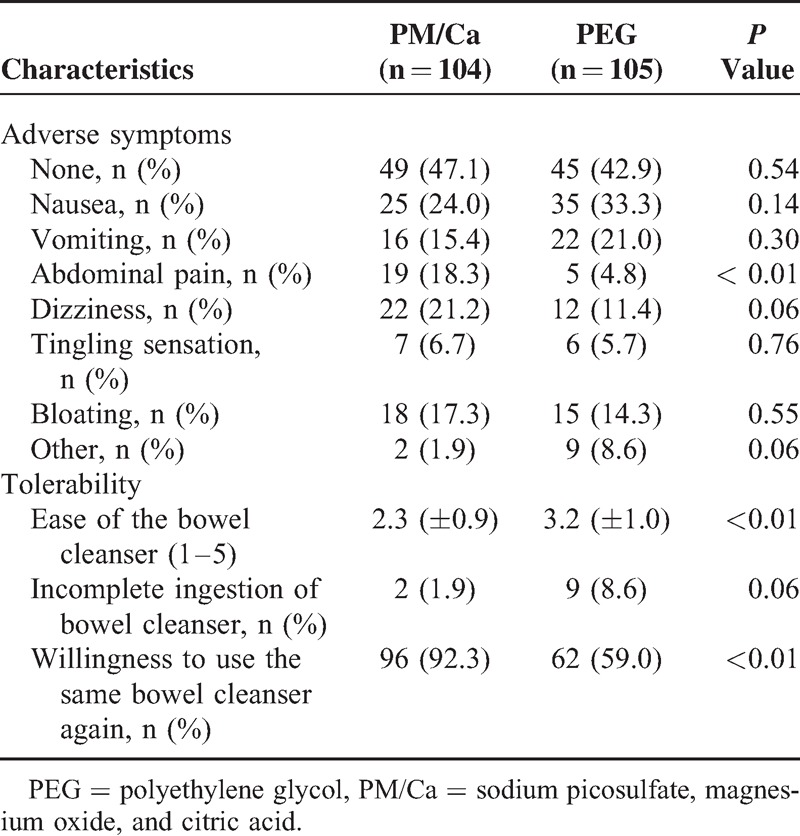
Adverse Symptoms and Tolerability of the Study Subjects

### Tolerability

The tolerability of the regimen as reported by study subjects is shown in Table 4. The mean ease of use score was 2.3 ± 0.9 in the PM/Ca group, which was superior to the score of 3.2 ± 1.0 in the PEG group (*P* < 0.01). The number of subjects who could not ingest the full dose was nonsignificantly higher in the PEG group. The study subjects who responded that they would use the same bowel cleanser again were higher in the PM/Ca group (92.3% vs 59.0%, *P* *<* 0.01).

### Polyp Detection Rate and Adenoma Detection Rate

The polyp detection rate and adenoma detection rate did not differ between the PM/Ca group and the PEG group: polyp detection rate, 49.5% versus 42.9%, respectively (*P* = 0.34) and adenoma detection rate, 39.8% versus 28.6%, respectively (*P* = 0.09).

## DISCUSSION

In the present study, we compared the bowel-cleansing efficacy of PM/Ca with that of PEG when bowel cleanser was administered on the same day of an afternoon colonoscopy. PM/Ca had similar bowel-cleansing efficacy and superior tolerability compared with PEG.

To obtain proper bowel-cleansing efficacy, the timing of the administration of the bowel cleanser is important.^1,16^ Split-dose PEG was reported to have superior bowel-cleansing efficacy than full-dose PEG given on the day before the examination.^17,18^ In 1 study, the probability of inadequate bowel preparation was higher when PEG was administered 1 day before the colonoscopy when the colonoscopy was performed in the afternoon compared with the morning.^19^ In that study, the interval between the bowel preparation and colonoscopy was longer in the study subjects who received afternoon colonoscopy compared with those who received morning colonoscopy. Hence, a longer interval between the bowel preparation and the start time of colonoscopy might be a risk factor for poor bowel preparation.^16^

Two studies compared administration of PEG 1 day before colonoscopy with PM/Ca given 1 day before the procedure and with a split-dose PM/Ca regimen.^5,6^ When the bowel cleanser was administered 1 day before colonoscopy, the bowel-cleansing efficacy did not differ between the PM/Ca and PEG groups.^5^ However, the other study found that the split-dose PM/Ca regimen produced better bowel-cleansing efficacy than did PEG administered 1 day before the colonoscopy.^6^ Even though the same bowel-cleansing agents were used in these 2 studies, the bowel-cleansing efficacy differed between dosing protocols.^5,6^ Therefore, in our study, to compare the 2 bowel cleansers without the possible confounding effect of different administration times, the study subjects ingested the bowel cleansers at the same time.

Patients who ingest the bowel cleanser in the morning and receive the colonoscopy in the afternoon on the same day have a shorter time for bowel evacuation compared with the split-dose regimen or previous-day administration. Rapid evacuation may increase the risk of electrolyte imbalance or hemodynamic instability. A few previous studies have shown that administration of the bowel cleanser on the same day of the afternoon colonoscopy improves bowel-cleansing efficacy.^11,12,20^ However, laboratory tests were not performed in these studies to prove the safety of such regimens. In our study, we checked the electrolyte concentrations and other laboratory test results after bowel preparation and found no significant electrolyte imbalance in the study subjects. After bowel preparation, the decrease in systolic blood pressure was greater in the PEG group compared with the PM/Ca group, but hemodynamic instability was not observed in any subjects. In our study, a higher percentage of study subjects complained of abdominal pain in the PM/Ca group (PM/Ca 18.3% vs PEG 4.8%, *P* < 0.01). The higher prevalence of abdominal pain in the PM/Ca group might reflect the effect of bisacodyl, which was administered only in this group. One study subject who cancelled the colonoscopy because of vomiting during the ingestion of PM/Ca changed to PEG as the bowel cleanser, but this person also vomited during ingestion of PEG. This suggests that vomiting was specific to this subject and did not reflect issues relating to the safety of PM/Ca.

Previous studies have shown that PM/Ca is well tolerated than PEG.^5–8^ Our findings are consistent with these earlier findings. Ease of use of the bowel cleanser was superior in the PM/Ca group (PM/Ca 2.3 ± 0.9 vs PEG 3.2 ± 1.0, *P* < 0.01), and the percentage of subjects willing to use the same bowel cleanser again was higher in the PM/Ca group (PM/Ca 92.3% vs PEG 59.0%, *P* *<* 0.01). Thus, the tolerability of PM/Ca was superior to that of PEG.

This study has some limitations to be addressed. First, it is a single-center trial. Although we included a large number of study subjects, more prospective studies are needed. Second, indication for colonoscopy differed between the 2 groups. However, we excluded the study subjects who had medical conditions such as constipation, liver cirrhosis, renal failure, and history of bowel surgery that were associated with inadequate bowel preparation.^21^ All study subjects were ambulatory and healthy without significant comorbidities. We believe that the difference did not affect the bowel-cleansing efficacy and tolerability. Third, blood tests were done only after the bowel preparation. An added blood test before the preparation would allow to investigate the change of the results.

In conclusion, the administration of PM/Ca on the morning of an afternoon colonoscopy was safe and showed similar bowel-cleansing efficacy and was well tolerated compared with PEG.

## Acknowledgments

*The authors would gratefully thank Suhyun Kim, Eunmi Lee, Jisun Oh, Jisun Seong, Jinhee Lee, Sol Kim, Suyeon Choi, Wonjin Choi, Youngju Kim, and Kyungha Hwang at Seoul St. Mary's Hospital for their support during the study. They would also thank Dr Hyun Woo Lim at the Catholic University of Korea for statistical consultation*.

## References

[1] Wexner SD Beck DE Baron TH A consensus document on bowel preparation before colonoscopy: prepared by a task force from the American Society of Colon and Rectal Surgeons (ASCRS), the American Society for Gastrointestinal Endoscopy (ASGE), and the Society of American Gastrointestinal and Endoscopic Surgeons (SAGES). *Surg Endosc* 2006; 20:1147–1160. 16763922 10.1007/s00464-006-0152-y

[2] Lebwohl B Kastrinos F Glick M The impact of suboptimal bowel preparation on adenoma miss rates and the factors associated with early repeat colonoscopy. *Gastrointest Endosc* 2011; 73:1207–1214. 21481857 10.1016/j.gie.2011.01.051 PMC3106145

[3] Hoy SM Scott LJ Wagstaff AJ Sodium picosulfate/magnesium citrate: a review of its use as a colorectal cleanser. *Drugs* 2009; 69:123–136. 19192941 10.2165/00003495-200969010-00009

[4] Lawrance I Willert R Murray K Bowel cleansing for colonoscopy: prospective randomized assessment of efficacy and of induced mucosal abnormality with three preparation agents. *Endoscopy* 2011; 43:412–418. 21547879 10.1055/s-0030-1256193

[5] Katz PO Rex DK Epstein M A dual-action, low-volume bowel cleanser administered the day before colonoscopy: results from the SEE CLEAR II study. *Am J Gastroenterol* 2013; 108:401–409. 23318484 10.1038/ajg.2012.441

[6] Rex DK Katz PO Bertiger G Split-dose administration of a dual-action, low-volume bowel cleanser for colonoscopy: the SEE CLEAR I study. *Gastrointest Endosc* 2013; 78:132–141. 23566639 10.1016/j.gie.2013.02.024

[7] Turner D Benchimol EI Dunn H Pico-Salax versus polyethylene glycol for bowel cleanout before colonoscopy in children: a randomized controlled trial. *Endoscopy* 2009; 41:1038–1045. 19967619 10.1055/s-0029-1215333

[8] Lawrance IC Willert RP Murray K A validated bowel-preparation tolerability questionnaire and assessment of three commonly used bowel-cleansing agents. *Dig Dis Sci* 2013; 58:926–935. 23095990 10.1007/s10620-012-2449-0

[9] Rahman A Vanner SJ Baranchuk A Serial monitoring of the physiological effects of the standard Pico-Salax(R) regimen for colon cleansing in healthy volunteers. *Can J Gastroenterol* 2012; 26:424–428. 22803016 10.1155/2012/757583 PMC3395442

[10] Shawki S Wexner SD Oral colorectal cleansing preparations in adults. *Drugs* 2008; 68:417–437. 18318561 10.2165/00003495-200868040-00003

[11] Matro R Shnitser A Spodik M Efficacy of morning-only compared with split-dose polyethylene glycol electrolyte solution for afternoon colonoscopy: a randomized controlled single-blind study. *Am J Gastroenterol* 2010; 105:1954–1961. 20407434 10.1038/ajg.2010.160

[12] Varughese S Kumar AR George A Morning-only one-gallon polyethylene glycol improves bowel cleansing for afternoon colonoscopies: a randomized endoscopist-blinded prospective study. *Am J Gastroenterol* 2010; 105:2368–2374. 20606677 10.1038/ajg.2010.271

[13] Longcroft-Wheaton G Bhandari P Same-day bowel cleansing regimen is superior to a split-dose regimen over 2 days for afternoon colonoscopy: results from a large prospective series. *J Clin Gastroenterol* 2012; 46:57–61. 22064553 10.1097/MCG.0b013e318233a986

[14] Hassan C Bretthauer M Kaminski MF Bowel preparation for colonoscopy: European Society of Gastrointestinal Endoscopy (ESGE) guideline. *Endoscopy* 2013; 45:142–150. 23335011 10.1055/s-0032-1326186

[15] Aronchick CA Lipshutz WH Wright SH A novel tableted purgative for colonoscopic preparation: efficacy and safety comparisons with Colyte and Fleet Phospho-Soda. *Gastrointest Endosc* 2000; 52:346–352. 10968848 10.1067/mge.2000.108480

[16] Eun CS Han DS Hyun YS The timing of bowel preparation is more important than the timing of colonoscopy in determining the quality of bowel cleansing. *Dig Dis Sci* 2011; 56:539–544. 21042853 10.1007/s10620-010-1457-1

[17] Park JS Sohn CI Hwang SJ Quality and effect of single dose versus split dose of polyethylene glycol bowel preparation for early-morning colonoscopy. *Endoscopy* 2007; 39:616–619. 17611916 10.1055/s-2007-966434

[18] Marmo R Rotondano G Riccio G Effective bowel cleansing before colonoscopy: a randomized study of split-dosage versus non-split dosage regimens of high-volume versus low-volume polyethylene glycol solutions. *Gastrointest Endosc* 2010; 72:313–320. 20561621 10.1016/j.gie.2010.02.048

[19] Sanaka MR Shah N Mullen KD Afternoon colonoscopies have higher failure rates than morning colonoscopies. *Am J Gastroenterol* 2006; 101:2726–2730. 17227519 10.1111/j.1572-0241.2006.00887.x

[20] Gurudu SR Ratuapli S Heigh R Quality of bowel cleansing for afternoon colonoscopy is influenced by time of administration. *Am J Gastroenterol* 2010; 105:2318–2322. 21048676 10.1038/ajg.2010.235

[21] Ness RM Manam R Hoen H Predictors of inadequate bowel preparation for colonoscopy. *Am J Gastroenterol* 2001; 96:1797–1802. 11419832 10.1111/j.1572-0241.2001.03874.x

